# Supplementary material for: Mycorrhizas drive the evolution of plant adaptation to drought
Source: Commun Biol. 2023 Mar 30;6:346. doi: 10.1038/s42003-023-04722-4 (PMC10063553; doi:10.1038/s42003-023-04722-4)
Supplement: Supplementary file 2 — Supplementary Information [file 42003_2023_4722_MOESM2_ESM.pdf]

Supplementary information for

Mycorrhizas drive the evolution of plant adaptation to drought

Marco Cosme

(marcosmeweb@gmail.com)

This PDF file includes:

Supplementary Fig. 1
Supplementary Table 1

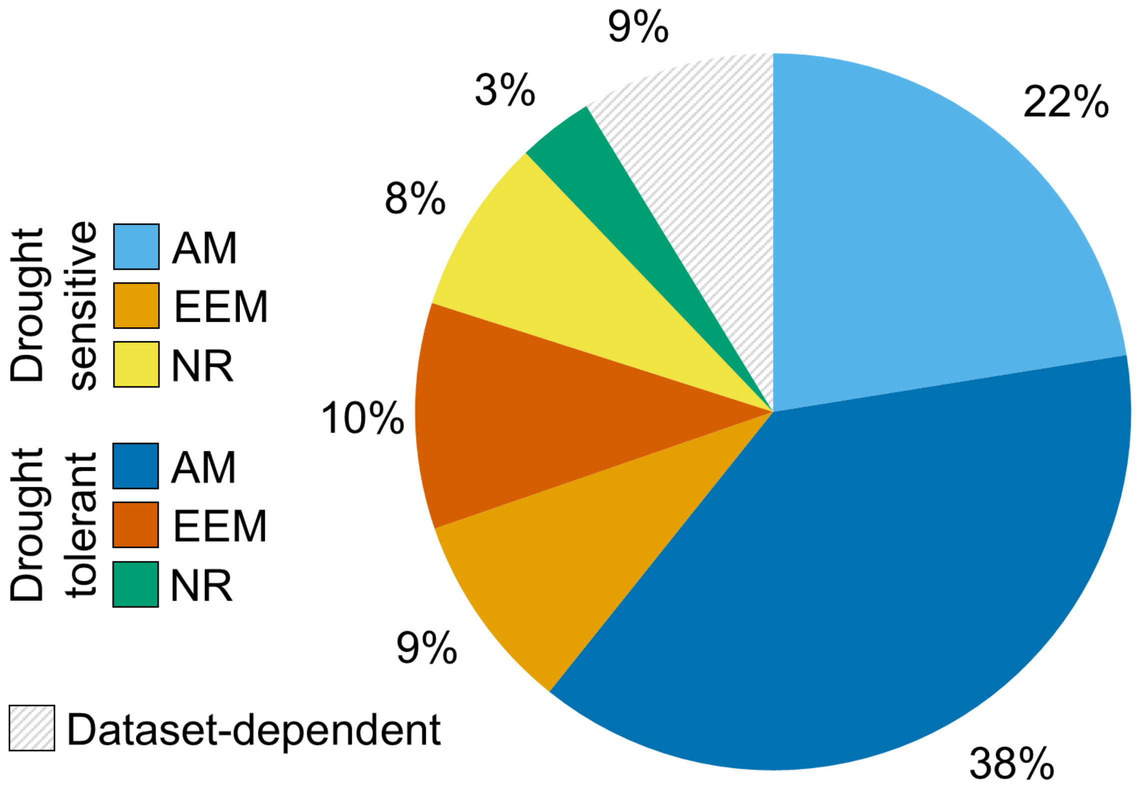

Supplementary Fig. 1: Percent distribution of mycorrhizal strategy and drought adaptation states.

The pie chart includes state data for 1,638 plant species (angiosperms and gymnosperms) colored according to their combined states of mycorrhizal strategy [AM, arbuscular mycorrhizal state; EEM, combined ectomycorrhizal and ericoid mycorrhizal state; NR, naked root state, i.e. non-mycorrhizal alone (dataset v5 and v6) or together with facultatively AM plants (dataset v1 to v4)] and drought adaptation (sensitive or tolerant), or a dataset-dependent state. The dataset-dependent state corresponds to assignments of mycorrhizal strategy and drought adaptation states varying according to dataset v1 to v6. The assembly of dataset v1 to v6 is summarized in Table 3 and described in the Methods.

Supplementary Table 1 Rates of evolutionary transitions from a combined mycorrhizal strategy and drought adaptation state to another as estimated by the model best fitted to each dataset version.

Hidden rate	Mycorrhizal strategy	Drought adaptation		Mycorrhizal strategy	Drought adaptation	Evolutionary transition rates (n° of transitions/Ma)																	
						Dataset v1			Dataset v2			Dataset v3			Dataset v4			Dataset v5			Dataset v6		
						Best	Lower CI	Upper CI	Best	Lower CI	Upper CI	Best	Lower CI	Upper CI	Best	Lower CI	Upper CI	Best	Lower CI	Upper CI	Best	Lower CI	Upper CI
**R1**	**AM**	**sensitive**	→	**AM**	**tolerant**	**15,0102324145092**	**15,0102324145092**	**17,3424010367719**	**6,8234734895013**	**5,3808676523914**	**7,8566975704230**	**13,6948208069163**	**13,6948208069163**	**18,3887585183864**	**2,1948551553455**	**2,0765814405262**	**2,1948551553455**	**6,9997851076192**	**5,0940088180811**	**71,7652926218442**	**6,9696099191187**	**5,8047691485696**	**18,0517693611206**
R1	AM	sensitive	→	EEM	sensitive	0,0003490751229	0,0002729099723	0,0003490751229	0,0000418488688	0,0000000009841	0,0004302991807	0,0000262122375	0,0000143213516	0,0001026275012	0,0000000010000	0,0000000000274	0,0000000019303	0,0002057800723	0,0000000021258	0,0003479593027	0,0002792103421	0,0000000011321	0,0010614107706
R1	AM	sensitive	→	NR	sensitive	0,0008276380883	0,0005728233079	0,0011038911648	0,0007384780292	0,0007384780292	0,0008119570634	0,0010450006738	0,0010165831441	0,0013521855758	0,0000000010000	0,0000000002006	0,0000000021763	0,0011140510433	0,0000000006126	0,0018092829541	0,0011404178026	0,0001360275527	0,0011404178026
**R1**	**AM**	**tolerant**	→	**AM**	**sensitive**	**7,5477224125858**	**7,5477224125858**	**9,0009475801222**	**3,3760262573811**	**2,8971809121337**	**3,5917122811357**	**5,6108202741940**	**5,6108202741940**	**7,8850835698804**	**1,3781914303773**	**1,2767119447199**	**1,3781914303773**	**6,9997851076192**	**5,0940088180811**	**71,7652926218442**	**6,9696099191187**	**5,8047691485696**	**18,0517693611206**
R1	AM	tolerant	→	EEM	tolerant	0,0004618452018	0,0002290125398	0,0005163526020	0,0009079602207	0,0008094841615	0,0009266698600	0,0005521349487	0,0005384115393	0,0007581632508	0,0000000010000	0,0000000006985	0,0000000021763	0,0002573510588	0,0000000008672	0,0007457345698	0,0001814158368	0,0000000012119	0,0007303845164
R1	AM	tolerant	→	NR	tolerant	0,0000000010000	0,0000000007853	0,0000000089202	0,0000000010000	0,0000000004278	0,0000000031500	0,0000000010000	0,0000000002104	0,0000000037613	0,0009883751259	0,0009268472211	0,0011499680443	0,0000000010000	0,0000000005900	0,0002935183308	0,0000000010000	0,0000000007007	0,0000000096757
R1	EEM	sensitive	→	AM	sensitive	0,0007933623592	0,0006376748772	0,0007933623592	0,0000600595324	0,0000000002991	0,0011294084895	0,0001078456690	0,0000000010343	0,0006665280445	0,0009655430086	0,0009001479162	0,0010087268541	0,0002057800723	0,0000000021258	0,0003479593027	0,0002792103421	0,0000000011321	0,0010614107706
**R1**	**EEM**	**sensitive**	→	**EEM**	**tolerant**	**57,0866902386243**	**48,5912589962842**	**65,5652111010726**	**174,6130998032230**	**149,2603376607140**	**202,8800764827220**	**100,0000000000000**	**90,2248468764835**	**116,7131872466280**	**40,0964859676181**	**32,5957734301285**	**43,4575845274555**	**73,8326087672995**	**33,7566240478965**	**105,8812989943900**	**73,8661872173341**	**38,2736703907384**	**219,0808321361410**
R1	EEM	sensitive	→	NR	sensitive	0,0000000010000	0,0000000009105	0,0013249448700	0,0000000010000	0,0000000006039	0,0000436577600	0,0000000010000	0,0000000005357	0,0000000089105	0,0000000010000	0,0000000008998	0,0000000020833	0,0000000010000	0,0000000004022	0,0001510912097	0,0000000010000	0,0000000006146	0,0001444615740
R1	EEM	tolerant	→	AM	tolerant	0,0001063795454	0,0000000010961	0,0001063795454	0,0008125807985	0,0004655631666	0,0009132700748	0,0003462193140	0,0000000012141	0,0004293294743	0,0000000010289	0,0000000004702	0,0000000012998	0,0002573510588	0,0000000008672	0,0007457345698	0,0001814158368	0,0000000012119	0,0007303845164
**R1**	**EEM**	**tolerant**	→	**EEM**	**sensitive**	**100,0000000000000**	**80,1791178868410**	**102,4770919833460**	**82,3656568432239**	**67,3327391319048**	**84,2016676635451**	**10,5863207218214**	**8,8127342711643**	**11,7615045183466**	**100,0000000000000**	**85,6126080519972**	**102,8593688631690**	**73,8326087672995**	**33,7566240478965**	**105,8812989943900**	**73,8661872173341**	**38,2736703907384**	**219,0808321361410**
R1	EEM	tolerant	→	NR	tolerant	0,0000000010000	0,0000000006933	0,0000000016227	0,0000000010000	0,0000000004598	0,0000000038168	0,0000000010000	0,0000000007104	0,0000000011598	0,0000000009747	0,0000000005330	0,0000000010064	0,0000000010000	0,0000000000657	0,0001566484589	0,0000000010000	0,0000000006039	0,0001265999833
R1	NR	sensitive	→	AM	sensitive	0,0000000010000	0,0000000003474	0,0000000020995	0,0000000009113	0,0000000004278	0,0000000066486	0,0000000010000	0,0000000007104	0,0000000023907	0,0000000010000	0,0000000007824	0,0007738851817	0,0011140510433	0,0000000006126	0,0018092829541	0,0011404178026	0,0001360275527	0,0011404178026
R1	NR	sensitive	→	EEM	sensitive	0,0000000010000	0,0000000006704	0,0000000013090	0,0006517535544	0,0003056499674	0,0007799754180	0,0006342389736	0,0004529134661	0,0009494572452	0,0000000010000	0,0000000009384	0,0000000019324	0,0000000010000	0,0000000004022	0,0001510912097	0,0000000010000	0,0000000006146	0,0001444615740
**R1**	**NR**	**sensitive**	→	**NR**	**tolerant**	**0,1066335745126**	**0,0661298048432**	**0,1066335745126**	**0,1262416415964**	**0,1064597016694**	**0,2615759651859**	**0,1294019985400**	**0,1248563596825**	**0,1306631364456**	**0,1206093416361**	**0,0947197121607**	**0,1301131841256**	**0,0815981466907**	**0,0597364288679**	**0,1027488506906**	**0,0811767340140**	**0,0811767340140**	**0,1485761364000**
R1	NR	tolerant	→	AM	tolerant	0,0000000010000	0,0000000002648	0,0000000018818	0,0000000010000	0,0000000001288	0,0004302991807	0,0000000010000	0,0000000002375	0,0000000054154	0,0000000010000	0,0000000008140	0,0000000018112	0,0000000010000	0,0000000005900	0,0002935183308	0,0000000010000	0,0000000007007	0,0000000096757
R1	NR	tolerant	→	EEM	tolerant	0,0000000010000	0,0000000005684	0,0000000052301	0,0000000010000	0,0000000002991	0,0009132714950	0,0000000010000	0,0000000010000	0,0000000030248	0,0000000010000	0,0000000002800	0,0000000012568	0,0000000010000	0,0000000000657	0,0001566484589	0,0000000010000	0,0000000006039	0,0001265999833
**R1**	**NR**	**tolerant**	→	**NR**	**sensitive**	**0,7498495911490**	**0,5152852426200**	**0,7498495911490**	**0,8602001065544**	**0,7961327663454**	**1,8378362284065**	**0,8981060052513**	**0,8981060052513**	**0,9722134895849**	**0,7873922443785**	**0,7873922443785**	**0,9511394888041**	**0,0815981466907**	**0,0597364288679**	**0,1027488506906**	**0,0811767340140**	**0,0811767340140**	**0,1485761364000**
**R2**	**AM**	**sensitive**	→	**AM**	**tolerant**	**0,0366920848535**	**0,0245135949179**	**0,0366920848535**	**0,0388090173548**	**0,0344788508381**	**0,0521323063676**	**0,0502824219686**	**0,0502824219686**	**0,0532689760045**	**0,0105484192083**	**0,0105484192083**	**0,0125742614836**	**0,0000000010000**	**0,0000000006126**	**0,0003479593027**	**0,0000000010000**	**0,0000000007375**	**0,0004428363627**
R2	AM	sensitive	→	EEM	sensitive	0,0000000010000	0,0000000010000	0,0000000016394	0,0000000010000	0,0000000004278	0,0000148306324	0,0000000010000	0,0000000002313	0,0000000049757	0,0000000010000	0,0000000000281	0,0000000014688	0,0000000010000	0,0000000004868	0,0003234773079	0,0000000010000	0,0000000000830	0,0008091672183
R2	AM	sensitive	→	NR	sensitive	0,0058483432567	0,0052852589093	0,0067934273123	0,0061947008406	0,0051084617075	0,0063709681814	0,0048650837535	0,0038796550044	0,0059991556979	0,0091985627085	0,0082065361260	0,0135974982305	0,0082668012088	0,0061083973930	0,0111766309736	0,0082481098625	0,0078487532713	0,0120720077626
**R2**	**AM**	**tolerant**	→	**AM**	**sensitive**	**0,0489583449820**	**0,0479735848480**	**0,0596604077385**	**0,0554485510397**	**0,0401364534980**	**0,0801056798049**	**0,0653851111476**	**0,0620305220289**	**0,0677085717907**	**0,0000000010000**	**0,0000000002536**	**0,0000000016942**	**0,0000000010000**	**0,0000000006126**	**0,0003479593027**	**0,0000000010000**	**0,0000000007375**	**0,0004428363627**
R2	AM	tolerant	→	EEM	tolerant	0,0007723557263	0,0006313620846	0,0010747654195	0,0000000009866	0,0000000002991	0,0000000056992	0,0010028382357	0,0005384115393	0,0010511648362	0,0018344391651	0,0018344391651	0,0022286451946	0,0009307564292	0,0000000007855	0,0017319467551	0,0009407334872	0,0000000017134	0,0012542654083
R2	AM	tolerant	→	NR	tolerant	0,0027151072318	0,0023731036713	0,0040246741695	0,0026771970502	0,0014565905671	0,0038761441228	0,0019919112258	0,0017637988161	0,0020242392198	0,0009108985259	0,0007012429860	0,0009342130093	0,0000000010000	0,0000000005551	0,0002935134715	0,0000000010000	0,0000000006804	0,0001266001888
R2	EEM	sensitive	→	AM	sensitive	0,0000000010000	0,0000000006631	0,0000000020785	0,0000000010000	0,0000000010000	0,0017676373393	0,0007155287654	0,0004557831856	0,0010309073271	0,0000000010000	0,0000000007824	0,0025691465823	0,0000000010000	0,0000000004868	0,0003234773079	0,0000000010000	0,0000000000830	0,0008091672183
**R2**	**EEM**	**sensitive**	→	**EEM**	**tolerant**	**0,9950528615947**	**0,9085612184972**	**1,0323001516767**	**26,5869569521618**	**25,9521911800217**	**34,0360455983783**	**34,6950494182448**	**32,3374418804419**	**38,3817146406614**	**7,5104418883213**	**7,5104418883213**	**9,2998488377663**	**0,0365772268929**	**0,0335832820957**	**0,0692585695122**	**0,0367190826322**	**0,0289315359080**	**0,0553850265033**
R2	EEM	sensitive	→	NR	sensitive	0,0000000010000	0,0000000002558	0,0000000013292	0,0000000010000	0,0000000004872	0,0000000041375	0,0000000010000	0,0000000008570	0,0000000017409	0,0000000010000	0,0000000003771	0,0000000010000	0,0000000010000	0,0000000005809	0,0002935167577	0,0000000010000	0,0000000002103	0,0004874503652
R2	EEM	tolerant	→	AM	tolerant	0,0000000010000	0,0000000008088	0,0000000016394	0,0000000010000	0,0000000008921	0,0000000038168	0,0000000010000	0,0000000000783	0,0000000018768	0,0000000010000	0,0000000003771	0,0000000017781	0,0009307564292	0,0000000007855	0,0017319467551	0,0009407334872	0,0000000017134	0,0012542654083
**R2**	**EEM**	**tolerant**	→	**EEM**	**sensitive**	**0,1573395326260**	**0,1389389635556**	**0,1695890529038**	**123,5457414767830**	**104,1303627980050**	**133,4770924912010**	**100,0000000000000**	**86,7354436120669**	**102,7709632884880**	**0,3947902765243**	**0,2978181579258**	**0,4115170356936**	**0,0365772268929**	**0,0335832820957**	**0,0692585695122**	**0,0367190826322**	**0,0289315359080**	**0,0553850265033**
R2	EEM	tolerant	→	NR	tolerant	0,0015959911772	0,0010837702928	0,0020237555180	0,0073235990603	0,0064417288511	0,0103249430373	0,0018019993260	0,0012867145349	0,0018019993260	0,0008673100896	0,0007026926997	0,0015045721058	0,0000000010000	0,0000000004868	0,0001929621468	0,0000000010000	0,0000000006506	0,0001265940134
R2	NR	sensitive	→	AM	sensitive	0,0000000010000	0,0000000002170	0,0000000022253	0,0000000010000	0,0000000008855	0,0000148299213	0,0000000010000	0,0000000002237	0,0000000054154	0,0179394855003	0,0170859001423	0,0275525429936	0,0082668012088	0,0061083973930	0,0111766309736	0,0082481098625	0,0078487532713	0,0120720077626
R2	NR	sensitive	→	EEM	sensitive	0,0023913422230	0,0013249447578	0,0026809644855	0,0000000010000	0,0000000009279	0,0000148267241	0,0000000010000	0,0000000006714	0,0000000012052	0,0000000010000	0,0000000000281	0,0000000015748	0,0000000010000	0,0000000005809	0,0002935167577	0,0000000010000	0,0000000002103	0,0004874503652
**R2**	**NR**	**sensitive**	→	**NR**	**tolerant**	**0,0211691418531**	**0,0171866990260**	**0,0211691418531**	**0,0235855704286**	**0,0157636913346**	**0,0413274636412**	**0,0231498524562**	**0,0231498524562**	**0,0283303432738**	**0,0215738966260**	**0,0160806529324**	**0,0333709098685**	**0,0000000010000**	**0,0000000004868**	**0,0002935138499**	**0,0000000010000**	**0,0000000000830**	**0,0006574337998**
R2	NR	tolerant	→	AM	tolerant	0,0120168131343	0,0114335638501	0,0154380796226	0,0119215052176	0,0100568749264	0,0120152448576	0,0125974545233	0,0082284242720	0,0125974545233	0,0032161851409	0,0022791754812	0,0034068481281	0,0000000010000	0,0000000005551	0,0002935134715	0,0000000010000	0,0000000006804	0,0001266001888
R2	NR	tolerant	→	EEM	tolerant	0,0000000010000	0,0000000008088	0,0000000016933	0,0000000010000	0,0000000008473	0,0000436593919	0,0000000010000	0,0000000008430	0,0000000030942	0,0014272893824	0,0012995264405	0,0018635369335	0,0000000010000	0,0000000004868	0,0001929621468	0,0000000010000	0,0000000006506	0,0001265940134
**R2**	**NR**	**tolerant**	→	**NR**	**sensitive**	**0,0000000010000**	**0,0000000008591**	**0,0000000089202**	**0,0000000009866**	**0,0000000000429**	**0,0000000075636**	**0,0017399866969**	**0,0000000012271**	**0,0040699344983**	**0,0008186267759**	**0,0000000008862**	**0,0025691465823**	**0,0000000010000**	**0,0000000004868**	**0,0002935138499**	**0,0000000010000**	**0,0000000000830**	**0,0006574337998**

Hidden rate corresponds to the level (R1 or R2) of the hidden phylogenetic factor that influences a given transition.
AM, arbuscular mycorrhizal state; EEM, combined ectomycorrhizal and ericoid mycorrhizal state; NR, naked root state, i.e. non-mycorrhizal alone (dataset v5 and v6) or together with facultatively AM (dataset v1 to v4).
Best, best estimation for the evolutionary transition rate; Lower CI and Upper CI correspond to the lower and upper value in the 95% confidence interval of each rate estimate.
Rows with text in bold inside the table correspond to evolutionary transitions between drought adaptation states within a given mycorrhizal strategy state
Rows with regular text inside the table correspond to transitions among mycorrhizal strategy states within a given drought adaptation state
The transition rates are the reconstructed numbers of ancestral transitions that have occurred over the past 325 million years of evolution, given here at a rate per mega-annum (Ma).
The assembly of dataset v1 to v6 is summarized in Table 3 and described in the Methods.
