# Supplementary material for: Mycorrhizas drive the evolution of plant adaptation to drought
Source: Commun Biol. 2023 Mar 30;6:346. doi: 10.1038/s42003-023-04722-4 (PMC10063553; doi:10.1038/s42003-023-04722-4)
Supplement: Supplementary file 3 — Reporting Summary [file 42003_2023_4722_MOESM3_ESM.pdf]

nature portfolio

Corresponding author(s): Marco Cosme
Last updated by author(s): Feb 23, 2023

Reporting Summary

Nature Portfolio wishes to improve the reproducibility of the work that we publish. This form provides structure for consistency and transparency in reporting. For further information on Nature Portfolio policies, see our Editorial Policies and the Editorial Policy Checklist.

Statistics

For all statistical analyses, confirm that the following items are present in the figure legend, table legend, main text, or Methods section.

n/a	Confirmed	
☐	☒	The exact sample size (n) for each experimental group/condition, given as a discrete number and unit of measurement
☐	☒	A statement on whether measurements were taken from distinct samples or whether the same sample was measured repeatedly
☐	☒	The statistical test(s) used AND whether they are one- or two-sided<br>*Only common tests should be described solely by name; describe more complex techniques in the Methods section.*
☐	☒	A description of all covariates tested
☒	☐	A description of any assumptions or corrections, such as tests of normality and adjustment for multiple comparisons
☐	☒	A full description of the statistical parameters including central tendency (e.g. means) or other basic estimates (e.g. regression coefficient) AND variation (e.g. standard deviation) or associated estimates of uncertainty (e.g. confidence intervals)
☒	☐	For null hypothesis testing, the test statistic (e.g. F, t, r) with confidence intervals, effect sizes, degrees of freedom and P value noted<br>*Give P values as exact values whenever suitable.*
☒	☐	For Bayesian analysis, information on the choice of priors and Markov chain Monte Carlo settings
☒	☐	For hierarchical and complex designs, identification of the appropriate level for tests and full reporting of outcomes
☒	☐	Estimates of effect sizes (e.g. Cohen's d, Pearson's r), indicating how they were calculated

Our web collection on statistics for biologists contains articles on many of the points above.

Software and code

Policy information about availability of computer code

Data collection	Data was collected and subset using the commercial software Excel of Microsoft Office 2019 and the open source software R version 4.1.1.
Data analysis	Data was analyzed using the open source software R version 3.6.2.

For manuscripts utilizing custom algorithms or software that are central to the research but not yet described in published literature, software must be made available to editors and reviewers. We strongly encourage code deposition in a community repository (e.g. GitHub). See the Nature Portfolio guidelines for submitting code & software for further information.

Data

Policy information about availability of data

All manuscripts must include a data availability statement. This statement should provide the following information, where applicable:

- Accession codes, unique identifiers, or web links for publicly available datasets
- A description of any restrictions on data availability
- For clinical datasets or third party data, please ensure that the statement adheres to our policy

The main Figures and main Tables are available in the article. The Supplementary Figure and Supplementary Table are available in the Supplementary Information of the article. The data generated and analyzed during the current study are provided as Supplementary Data and are available in the Dryad digital repository at https://doi.org/10.5061/dryad.3ffbg79nx.

Human research participants

Policy information about studies involving human research participants and Sex and Gender in Research.

Reporting on sex and gender	This information has not been collected.
Population characteristics	See above.
Recruitment	See above.
Ethics oversight	See above.

Note that full information on the approval of the study protocol must also be provided in the manuscript.

Field-specific reporting

Please select the one below that is the best fit for your research. If you are not sure, read the appropriate sections before making your selection.

☐ Life sciences ☐ Behavioural & social sciences ☒ Ecological, evolutionary & environmental sciences

For a reference copy of the document with all sections, see nature.com/documents/nr-reporting-summary-flat.pdf

Ecological, evolutionary & environmental sciences study design

All studies must disclose on these points even when the disclosure is negative.

Study description	This study uses a phylogenetic comparative approach based on Hidden Markov Models of varying evolutionary complexity to test for correlation evolution between two plant characters and to estimate the rates of evolutionary transitions among the character states. One character has three levels and the other two levels. The experimental unit is the plant species and a total of 1,638 species were considered. The number of plant species per character state combination varied from 20 to 408 depending on state and dataset version. A total of six partially different dataset versions were analyzed.
Research sample	The research sample is a group of globally distributed plant species (angiosperms and gymnosperms) that reflect a large sample for which there is available combined data on mycorrhizal strategy, drought adaptation, and phylogeny. Data was collected from existing datasets provided by the Global Biodiversity Information Facility (https://doi.org/10.15468/dl.5mab9f), TRY – Plant Trait Database (Kattge et al. 2020. Glob Change Biol 26, 119-188), FungalRoot (Soudzilovskaia et al. 2020. New Phytol 227, 955-966), Bueno et al (2018) Mycorrhiza 29, 1-11, and Smith and Brown (2018) Am J Bot 105, 302-314.
Sampling strategy	Data were obtained from curated datasets in order to generate a large global sample of plant species with combined data on mycorrhizal strategy, drought adaptation, and phylogeny. Six partially different datasets were generated to account for data uncertainty. For each dataset version, the phylogenetic imbalance ratio (Gardner and Organ 2022 Syst Biol 70, 1061-1075) was calculated to determine the adequacy of the sample to test for correlated evolution. Finally, the 95% confidence intervals for each estimated rate parameter were determined.
Data collection	All data in existing datasets were downloaded by the author from an online database and from literature supporting information. These data were processed by the author using Excel and R software.
Timing and spatial scale	The data used in this study is based on multiple records collected over a century and reflect functional traits of extant plant species.
Data exclusions	No data were excluded from the study.
Reproducibility	18 different Hidden Markov Models were tested in this study. Each model was independently run on six partially different dataset versions, each one performed with five repeated starts, resulting in a total of 810 independent optimization exercises. The 95% confidence intervals for each rate parameter estimate were determined with a dentist approach using 5,000 points sampled around a specified distance from the maximum likelihood estimates.
Randomization	Allocation to groups was based on species traits and therefore non-randomly dependent on phylogenetic relationships. To account for this, the study employed Hidden Markov Models that consider hidden rate categories accounting for unobserved phylogenetic factors influencing state reconstruction and rate estimates.
Blinding	Data was assigned to each species using a complete blind approach, based solely on automated species name matches. Selection of estimated parameters was based on the goodness of fit (sample size-corrected Akaike information criterion), first among the five repeated starts of each model run, and second among the 18 different models of evolution.

Did the study involve field work? ☐ Yes ☒ No

Reporting for specific materials, systems and methods

We require information from authors about some types of materials, experimental systems and methods used in many studies. Here, indicate whether each material, system or method listed is relevant to your study. If you are not sure if a list item applies to your research, read the appropriate section before selecting a response.

Materials & experimental systems

n/a	Involved in the study
☒	☐ Antibodies
☒	☐ Eukaryotic cell lines
☒	☐ Palaeontology and archaeology
☒	☐ Animals and other organisms
☒	☐ Clinical data
☒	☐ Dual use research of concern

Methods

n/a	Involved in the study
☒	☐ ChIP-seq
☒	☐ Flow cytometry
☒	☐ MRI-based neuroimaging
